# Europium (III) as a Circularly Polarized Luminescence Probe of DNA Structure

**DOI:** 10.1038/s41598-018-37680-7

**Published:** 2019-01-31

**Authors:** Tao Wu, Petr Bouř, Valery Andrushchenko

**Affiliations:** Institute of Organic Chemistry and Biochemistry, Czech Academy of Sciences, Flemingovo náměstí 2, 16610 Prague 6, Czech Republic

## Abstract

We report as a proof-of-concept the first application of circularly polarized luminescence (CPL) measured with a Raman optical activity (ROA) spectrometer to differentiate several DNA structures without need of sensitizing complexes. The ROA/CPL approach provides sufficiently high CPL intensity to use hydrated Eu^3+^ ions, thus avoiding DNA structural changes associated with binding of sensitizers and overcoming the sensitizer quenching issue. We showed that deoxyguanosine monophosphate (dGMP), single- and double-stranded DNA provide different CPL spectra, which could be used for their discrimination. Our results demonstrate that ROA/CPL method is a promising approach to measure CPL spectra of complex biomolecules when the use of sensitizers is not possible. The method can be extended to other biomolecules, such as proteins, lipids, sugars, etc.

## Introduction

Circularly polarized luminescence (CPL) of lanthanides is extremely sensitive to local molecular environment. Compared to the total luminescence (TL)^1–7^, CPL provides additional information on chirality and conformation of studied systems^8,9^. Because the nearly forbidden transitions within the *f* electronic states of lanthanides normally provide only a weak intensity, sensitizing organic complexes must be used to increase the emission^9–12^.

However, application of CPL to DNA studies using commercial CPL spectrometers has been so far unsuccessful due to low signal intensity and quenching of the luminescence of sensitizing agents upon their binding to DNA^10–12^. Furthermore, the sensitizers can distort the DNA structure upon intercalation^11^.

In the present work we demonstrate a proof-of-concept that lanthanide CPL can be successfully measured for DNA using Raman optical activity (ROA) spectrometer (ROA/CPL method^13^). The method provides sufficiently high intensity of the CPL signal to avoid sensitizing complexes and use bare (hydrated-only) lanthanide ions at relatively low concentrations. Eu^3+^ is the most suitable metal because its excitation energy (^5^D_0_ $\to$ ^7^F_1_) is close to the 532 nm wavelength of the excitation laser used in the ROA spectrometer. Different DNA model systems tested in the present work provided distinct CPL spectra, allowing for their discrimination. Vibrational ROA signal (difference in scattering of the right and left circularly polarized light) is present in the spectrum as well, generally enabling measurement of four spectral types (TL, CPL, Raman and ROA spectra) in a single experiment, which makes the approach very attractive for structural studies of biomolecules. It has been recently shown that the method can be successfully used for studies of sugars, amino acids and proteins^14,15^. In the future, we plan to investigate the effect of DNA base content, conformation, number of strands, salt content, pH and temperature on the CPL signal.

## Results and Discussion

### General Features of ROA/CPL Spectra of Eu^3+^ Complexed with DNA Model Systems

**Figure 1:**
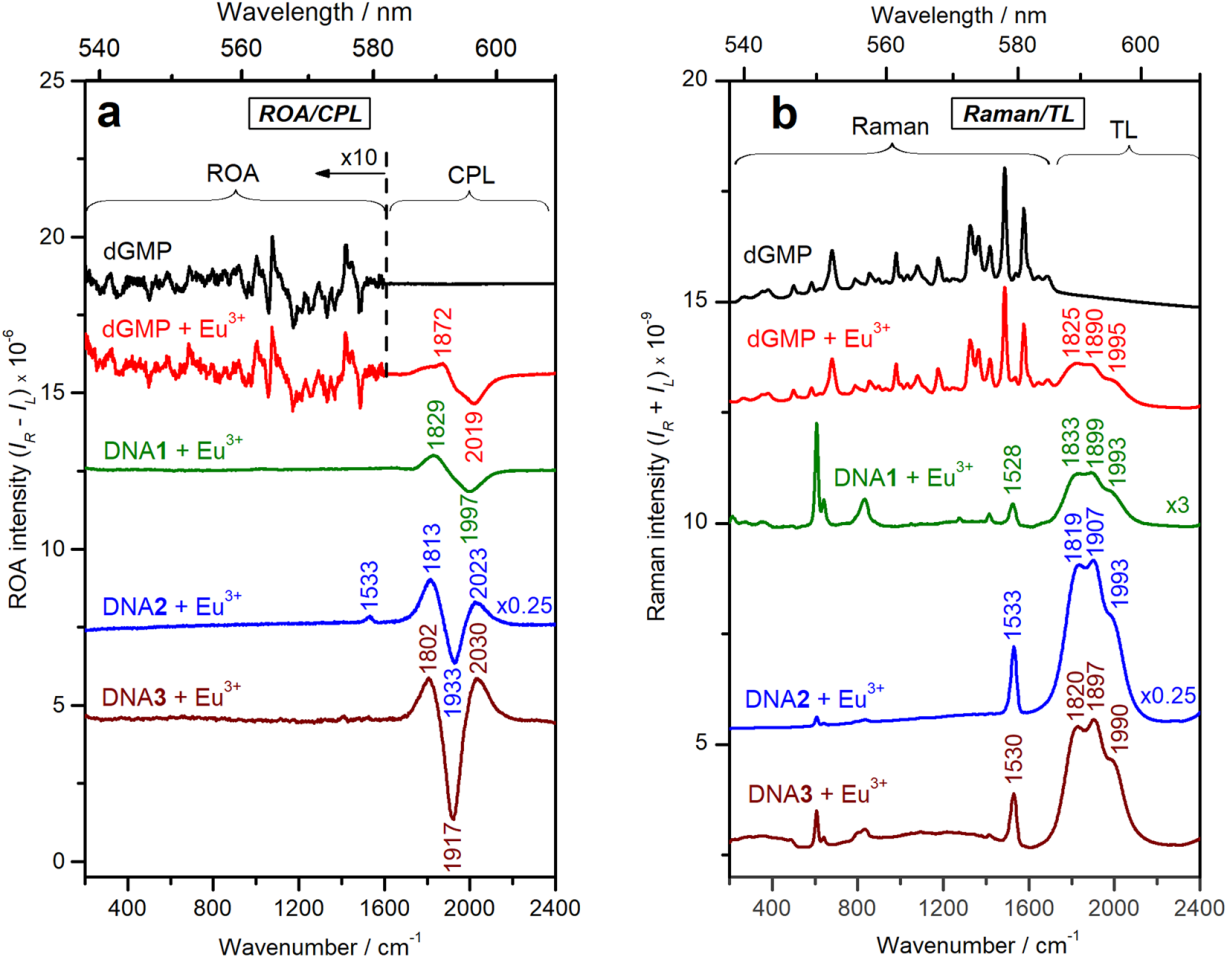
ROA/CPL (**a**) and Raman/TL (**b**) spectra of blank dGMP and dGMP, DNA**1**, DNA**2** and DNA**3** complexes with EuCl_3_. The spectral ranges corresponding to mainly ROA/Raman and mainly CPL/TL signal are indicated with curly brackets. *I*_*L*_ and *I*_*R*_ represent the corresponding intensities of left- and right-circularly polarized emission or Raman scattering. The details on the sample concentrations are provided in Supplementary Information.

CPL spectra of Eu^3+^ aqua-ion bound to dGMP and three model DNA systems are presented in Fig. 1. The CPL band shapes significantly vary, indicating different local chiral environment of Eu^3+^ and suggesting different binding modes and sites.

Further analysis can help us to understand the spectra better. Lanthanide luminescence can be clearly distinguished from the vibrational Raman signal by application of an external magnetic field (Supplementary Fig. S1), which also helps to further resolve and assign the luminescence bands (Supplementary Table S1). Europium binding to the DNA samples changes the spherical (on average) Eu^3+^ symmetry and its total luminescence (cf. Figure 1b and Supplementary Fig. S1). Particularly, a very weak band around 1521 cm^−1^ (579 nm) in EuCl_3_ water solution (^5^D_0_ $\to$ ^7^F_0_) (Supplementary Fig. S1), known to be especially symmetry-sensitive^16^, increases in intensity and shifts to cca. 1530 cm^−1^ upon metal binding to DNA (Fig. 1b). Table 1 outlines assignments of the main luminescence bands observed in Eu^3+^-DNA complexes. The highest intensity bands around 1800–2000 cm^−1^ (588–595 nm) originate from degenerate sublevels of ^5^D_0_ $\to$ ^7^F_1_ europium transition^17^. The TL band around 1530 cm^−1^ (^5^D_0_ $\to$ ^7^F_0_ transition) does not show measurable CPL except for the case of DNA**2** (1533 cm^−1^). Although the bands above 2450 cm^−1^ (>610 nm) (^5^D_0_ $\to$ ^7^F_2_ transition) are not accessible with our instrument, they could be measured with extended-range custom-made ROA spectrometers^18,19^, thus this is not a general limitation of the method.

**Table 1:** Positions of the luminescence bands (Raman shift *δ* in cm^−1^ and wavelength *λ* in nm) observed in CPL spectra of dGMP and DNA complexes with Eu^3+^.

dGMP		DNA1		DNA2		DNA3		Eu^3+^ transition
δ	λ	δ	λ	δ	λ	δ	λ	
>2450	>610	>2450	>610	>2450	>610	>2450	>610	^5^D_0_ $\to$ ^7^F_2_
2019	596	1997	595	2023	596	2030	596	^5^D_0_ $\to$ ^7^F_1_
1995	595	1993	595	1993	595	1990	595	″
				1933	593	1917	592	″
1890	591	1899	592	1907	592	1897	592	″
1872	591	1829	589	1813	589	1802	588	″
1825	589	1833	589	1819	589	1820	589	^5^D_0_ $\to$ ^7^F_1_
		1528	579	1533	579	1530	579	^5^D_0_ $\to$ ^7^F_0_

### ROA/CPL Spectra of Eu^3+^ Complexes with dGMP

**Figure 2:**
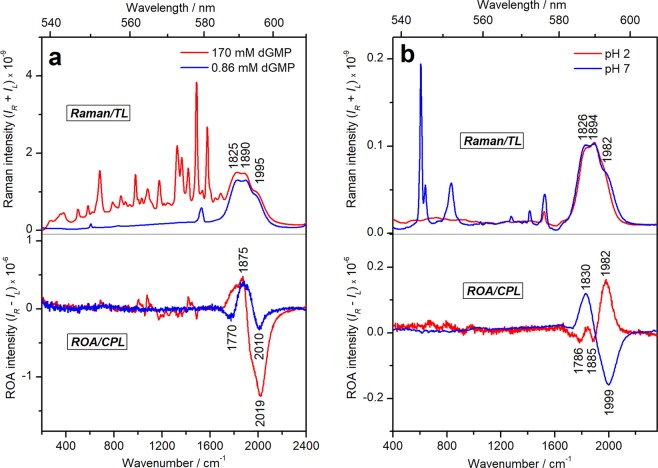
(**a**) Raman/TL and ROA/CPL spectra of dGMP-Eu^3+^ complexes at 170 mM of dGMP + 0.19 mM of EuCl_3_ (0.001 [Eu^3+^]/[dGMP]) (red) and at 0.86 mM of dGMP + 0.4 mM of EuCl_3_ (0.47 [Eu^3+^]/[dGMP]) (blue). (**b**) Raman/TL and ROA/CPL spectra of Eu^3+^– DNA**1** complexes at pH 2 (red) (3.2 mM (P) + 0.24 mM of EuCl_3_; 0.075 [Eu^3+^]/[P]) and pH 7 (blue) (2.24 mM (P) + 0.11 mM EuCl_3_; 0.05 [Eu^3+^]/[P]).

Despite the very low [Eu^3+^]/[dGMP] ratio of 0.001, the metal ion binding to dGMP is easily detectable by the presence of a strong CPL signal in the region of 1700–2200 cm^−1^ (585–603 nm). The TL/CPL spectra are complemented by the rich Raman and ROA spectra in the 200–1600 cm^−1^ range. The (+/−) CPL pattern observed at [Eu^3+^]/[dGMP] = 0.001 (170 mM of dGMP) changed to (−/+/−) pattern at [Eu^3+^]/[dGMP] = 0.47 (0.86 mM of dGMP), indicating different chiral environment of the lanthanide ion (Fig. 2a). At low dGMP concentrations mainly monomeric metal-dGMP complexes are expected, while at high concentrations dGMP molecules can form supramolecular associations templated by Eu^3+^ ions. Self-association and polymerization of the purine nucleotides through lanthanide-mediated bridging of the phosphate groups was suggested previously^2,3,20^. Indeed, infrared (IR) spectra obtained at high dGMP concentration (100 mM of dGMP) demonstrated large changes in the phosphate region (1150–950 cm^−1^) and only minor changes in the nitrogen base region (1750–1500 cm^−1^), suggesting extensive direct metal-phosphate coordination with minimal indirect metal-base binding at these conditions (Supplementary Fig. S2).

### ROA/CPL Spectra of Eu^3+^ Complexes with Single-stranded DNA

CPL spectrum of DNA**1** has similar pattern as that of dGMP ((+/−) couplet around 1900 cm^−1^ (592 nm)), although the signal is shifted to lower wavenumbers by about 25 cm^−1^, suggesting comparable but not identical Eu^3+^ binding environments (Fig. 1a). We used relatively low DNA concentration (2.24 mM (P)) to explore concentration requirements of the ROA/CPL method, therefore neither ROA nor Raman signal was observed at these conditions (the Raman bands around 600–850 cm^−1^ (Fig. 1b) come from the cacodylate buffer). Recording of Raman and ROA spectra of DNA**1** would require a concentration of 100–150 mM (P)^21^, which is much higher than that needed for the CPL measurement. The IR and vibrational circular dichroism (VCD) spectra indicate that DNA**1** has a single-stranded structure preserving local helicity (Supplementary Fig. S3). Evidence for that comes from the C = O vibrations of the bases at 1662 cm^−1^ and sugar C–O stretching vibration at 1060 cm^−1^, accompanied by the B-form IR marker bands at 939, 896 and 837 cm^−1^ and well-defined VCD couplets^22–26^. At [Eu^3+^]/[P] = 0.05 (also used in the ROA/CPL experiment), there are only slight changes in the IR and VCD spectra characteristic for a minor extent of metal ion binding to both the phosphate groups and the bases (mainly guanine base), while the overall DNA structure remains unchanged. Lack of extensive base pairing in short single-stranded DNA**1** could lead to somewhat similar binding sites available both in DNA**1** and in dGMP, possibly resulting in a similar (+/−) CPL pattern. On the other hand, the observed differences in the CPL signal might arise from the diester nature of the phosphate and from larger participation of the bases in the metal binding in DNA**1** compared to dGMP.

Very interesting is the CPL sensitivity to pH changes shown in Fig. 2b. The main pattern of CPL band changes from the (+/−) couplet at pH 7 to a (*−*/*−*/+) shape at pH 2. Most of DNA binding sites are protonated at such a low pH^27^, which, clearly, drastically affects the Eu^3+^ binding and CPL spectra.

### ROA/CPL Spectra of Eu^3+^ Complexes with Double-stranded DNA

CPL spectra for both DNA**2** and DNA**3** have similar (+/−/+) pattern, which, however, is different from the (+/−) couplet shape observed for the dGMP and DNA**1** complexes (Fig. 1a). Analogous (+/−/+) CPL pattern was observed for a sensitizing Eu^3+^ complex bound to poly(dGdC) and was assigned to predominantly metal-phosphate interactions^10^. The IR and VCD spectra confirm the double-helical B-conformation for DNA**2** (Supplementary Fig. S4). Evidence for that comes from the C = O vibrations of the bases at 1680 cm^−1^ and sugar C–O stretching vibration at 1054 cm^−1^, accompanied by the B-form IR marker bands at 939, 896 and 839 cm^−1^ and strong VCD couplets characteristic for double-stranded B-DNA^22–26^. The electronic circular dichroism (ECD) spectrum of DNA**3** is also characteristic for the double-stranded structure in B-conformation (Supplementary Fig. S4)^28^. The IR/VCD spectra show very little changes upon adding Eu^3+^ even at twice as high [Eu^3+^]/[P] ratio as that used in the ROA/CPL experiment (Supplementary Fig. S4), suggesting mainly indirect water-mediated metal ion binding to double-stranded DNA and absence of a substantial distortion of DNA structure. The ECD spectral changes observed for Eu^3+^– DNA**3** complex are attributed to a partial DNA compaction (condensation) rather than to changes in the secondary structure (Supplementary Fig. S4)^3,26^. The double-stranded conformation of DNA**2** and DNA**3** results in different metal binding sites compared to dGMP and short single-stranded DNA**1**, which might lead to the observed differences in the CPL pattern.

The dissymmetry factor *g* (*g* = 2 × (*I*_*L*_ *– I*_*R*_)/(*I*_*L*_ + *I*_*R*_), where *I*_*L*_ and *I*_*R*_ represent the corresponding intensities of left- and right-circularly polarized emission) allows to compare the intensity of the CPL signal for different samples. Because CPL signal arises only from the Eu^3+^ bound to a chiral host, its intensity expressed as dissymmetry factor could allow to estimate the extent of the lanthanide binding. The *g* values provided in Table 2 and plotted in Supplementary Fig. S5 suggest more extensive Eu^3+^ binding to dGMP and single-stranded DNA**1**, as opposite to the double-stranded DNA**2** and DNA**3**, in agreement with previous studies^5–7^.

**Table 2:** Maximum dissymmetry factor (*g*_*max*_) values observed for the dGMP and DNA complexes with Eu^3+^.

	dGMP	DNA1	DNA2	DNA3
g_max_	7.0×10^−3^	7.2×10^−3^	8.6×10^−4^	2.4×10^−3^
wavenumber, cm^−1^	2044	2031	1935	1924

## Conclusions

We demonstrated that the ROA/CPL method enables sensitive probing of DNA structure using europium (III) aqua-ions, without the need of sensitizing agents. Despite certain current limitations not related to the method *per se* (relatively narrow usable wavenumber/wavelength range, fixed excitation laser wavelength), it could allow for CPL studies of various DNA structures as well as other complex biomolecules, especially when usage of sensitizing lanthanide complexes is not possible. Furthermore, if higher sample concentrations are used, the Raman and ROA spectra of the host molecule could be obtained simultaneously with the CPL and TL spectra, providing additional structural data. Systematic calibration and theoretical rationalization of the experimental CPL spectra based on quantum chemical computations, which are currently underway in our laboratory, will significantly enhance the information obtained by the method in the future. We believe that the methodology based on induced lanthanide chirality could offer numerous applications to studies of various biological systems.

## Materials and Methods

### Materials

Sodium salts of herring sperm DNA (crude oligonucleotides, ~50–150 bp; referred to as “DNA**1**”), herring testes DNA (~7 000 bp; “DNA**2**”) and salmon sperm DNA (~20 000 bp; “DNA**3**”) and deoxyguanosine monophosphate (dGMP) as well as EuCl_3_·6H_2_O and NaCl salts, sodium cacodylate and D_2_O (99.9%) were purchased from Sigma-Aldrich. The DNA length was determined by gel electrophoresis (Supplementary Fig. S6). Cacodilic buffer containing 40 mM of sodium cacodylate and 60 mM NaCl (pH 6.5–7.0) was prepared with MilliQ water (18 MΩ·cm impedance) or D_2_O. DNA concentration is indicated in moles of DNA phosphate groups (P). All concentrations refer to final values in the spectroscopic cell/cuvette.

### ROA/CPL Spectra Measurements

EuCl_3_ (0.1–2 mM) was added to dGMP and DNA dissolved in cacodilic buffer. Raman/TL and ROA/CPL spectra were acquired with a Chiral Raman-2X ROA spectrometer (BioTools Inc., Jupiter, USA) using 532 nm laser excitation, 7 cm^−1^ resolution, 900 mW laser power at the sample, and 2 h collection time. All experiments were performed at room temperature. To assign the europium transitions, magnetic ROA/CPL (MROA/MCPL) spectra of EuCl_3_ were measured following previous routine^29^. Water baseline was subtracted from the Raman/TL spectra. The spectra are reported both in the wavenumber scale (shift from the 532 nm excitation, i.e. 200–2400 cm^−1^) as is usual in Raman spectroscopy (bottom axes) and in the wavelength scale (538–610 nm) conventional for CPL spectroscopy (top axes). Details on the sample concentrations used for the spectra shown in Fig. 1 and description of other experimental procedures are provided in Supplementary Information.

## Supplementary information

Supplementary Information
