# Supplementary material for: Europium (III) as a Circularly Polarized Luminescence Probe of DNA Structure
Source: Sci Rep. 2019 Jan 31;9:1068. doi: 10.1038/s41598-018-37680-7 (PMC6355874; doi:10.1038/s41598-018-37680-7)
Supplement: Supplementary file 1 — Supplementary Information [file 41598_2018_37680_MOESM1_ESM.pdf]

Supplementary Information

Europium (III) as a Circularly Polarized Luminescence Probe of DNA Structure

Tao Wu, Petr Bouř and Valery Andrushchenko*

Institute of Organic Chemistry and Biochemistry, Czech Academy of Sciences

Flemingovo náměstí 2, 16610 Prague 6, Czech Republic

* Correspondence and requests for materials should be addressed to V.A.

email: andrushchenko@uochb.cas.cz

Experimental details

Sample concentrations used for acquisition of the ROA/CPL spectra shown in Figure 1.

The sample concentrations used for the spectra shown in Figure 1 are following (from top to bottom): blank dGMP (200 mM); dGMP (170 mM) + $EuCl_3$ (0.19 mM) (0.001 $[Eu^{3+}]/[dGMP]$); DNA**1** (2.24 mM (P)) + $EuCl_3$ (0.11 mM) (0.05 $[Eu^{3+}]/[P]$); DNA**2** (9.6 mM (P)) + $EuCl_3$ (1 mM) (0.1 $[Eu^{3+}]/[P]$); DNA**3** (9.5 mM (P)) + $EuCl_3$ (0.4 mM) (0.04 $[Eu^{3+}]/[P]$).

Infrared/vibrational circular dichroism (IR/VCD) spectra measurements

The deuterium exchange for DNA and dGMP was achieved by lyophilizing and redissolving the samples three times in D_2O. The samples were contained in a demountable cell (International Crystal Laboratories, Inc., Garfield, USA) composed of two BaF_2 windows separated by a 50 μm Teflon spacer. The DNA or dGMP stock solution (30 μL) was deposited on the bottom cell window and $EuCl_3$ solution (15 μL) added dropwise with continuous stirring. Then the sample was covered by the top window. VCD spectra were measured with a Chiral IR-2X VCD spectrometer (BioTools, Inc., Jupiter, USA) as blocks of 1560 scans (~ 30 min) at 4 cm^{-1} resolution. In total 36 blocks were acquired and subsequently averaged to increase S/N ratio. The measurements were done at room temperature (temperature at the sample was ~27 °C). The solvent spectra measured at identical conditions were subtracted from the sample spectra.

Electronic circular dichroism (ECD) spectra measurements

$EuCl_3$ (0.4 mM) was added to DNA**3** (10 mM (P)) dissolved in cacodilic buffer. The sample was placed in a rectangular quartz cell with path length of 10 μm. The spectra were recorded at 20 nm/min scanning speed, 4 s response time and 0.1 nm resolution using a Jasco J-815 spectropolarimeter (JASCO Corporation, Tokyo, Japan). A total of 3 scans were accumulated and averaged in 185–320 nm range. The solvent spectrum measured at identical conditions was subtracted from the sample spectra. The experiments were performed at room temperature.

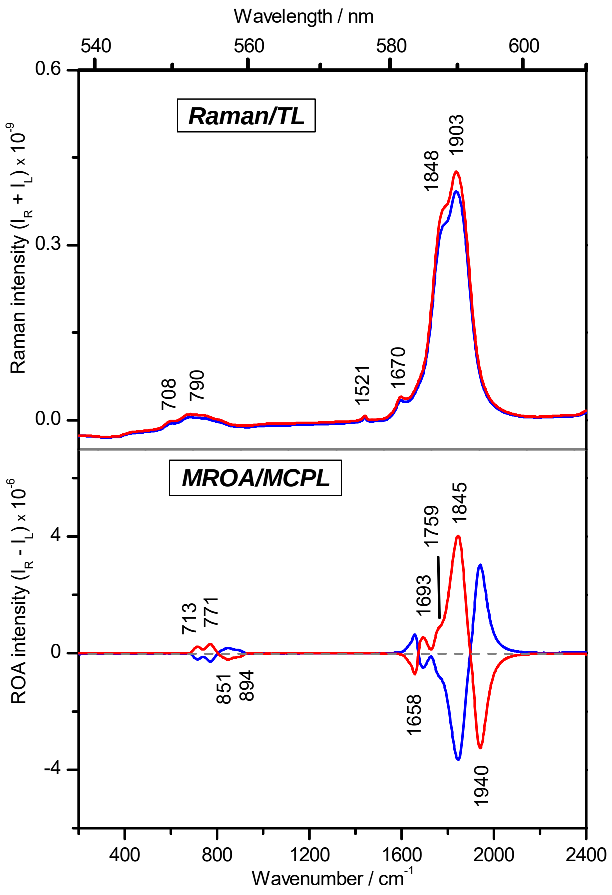

Figure S1. Raman/TL and MROA/MCPL spectra of $EuCl_3$ water solution (0.59 M) for two magnet orientations.

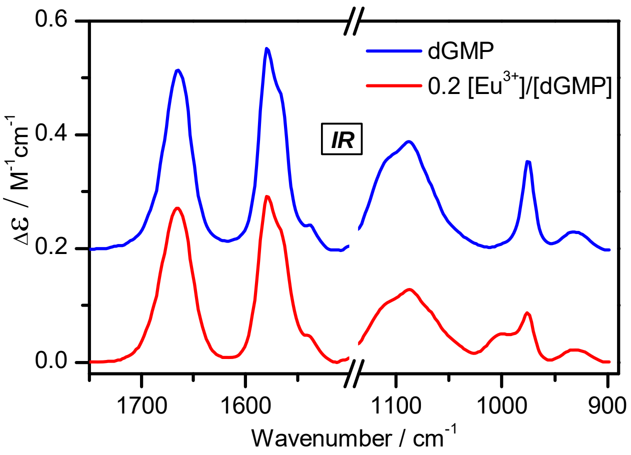

Figure S2. IR spectra of blank dGMP (100 mM) and in the presence of 20 mM of $EuCl_3$ (0.2 $[Eu^{3+}]$/[dGMP]). The IR spectra are vertically shifted for clarity.

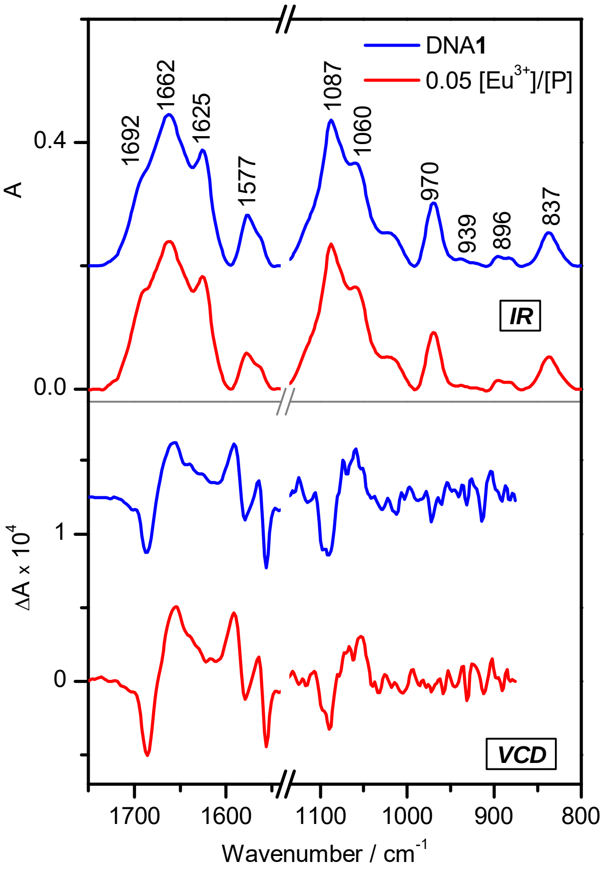

Figure S3. IR (top) and VCD (bottom) spectra of blank DNA**1** (96 mM (P)) and in the presence of 4.8 mM of $EuCl_3$ (0.05 [Eu^{3+}]/[P]). The IR and VCD spectra are vertically shifted for clarity.

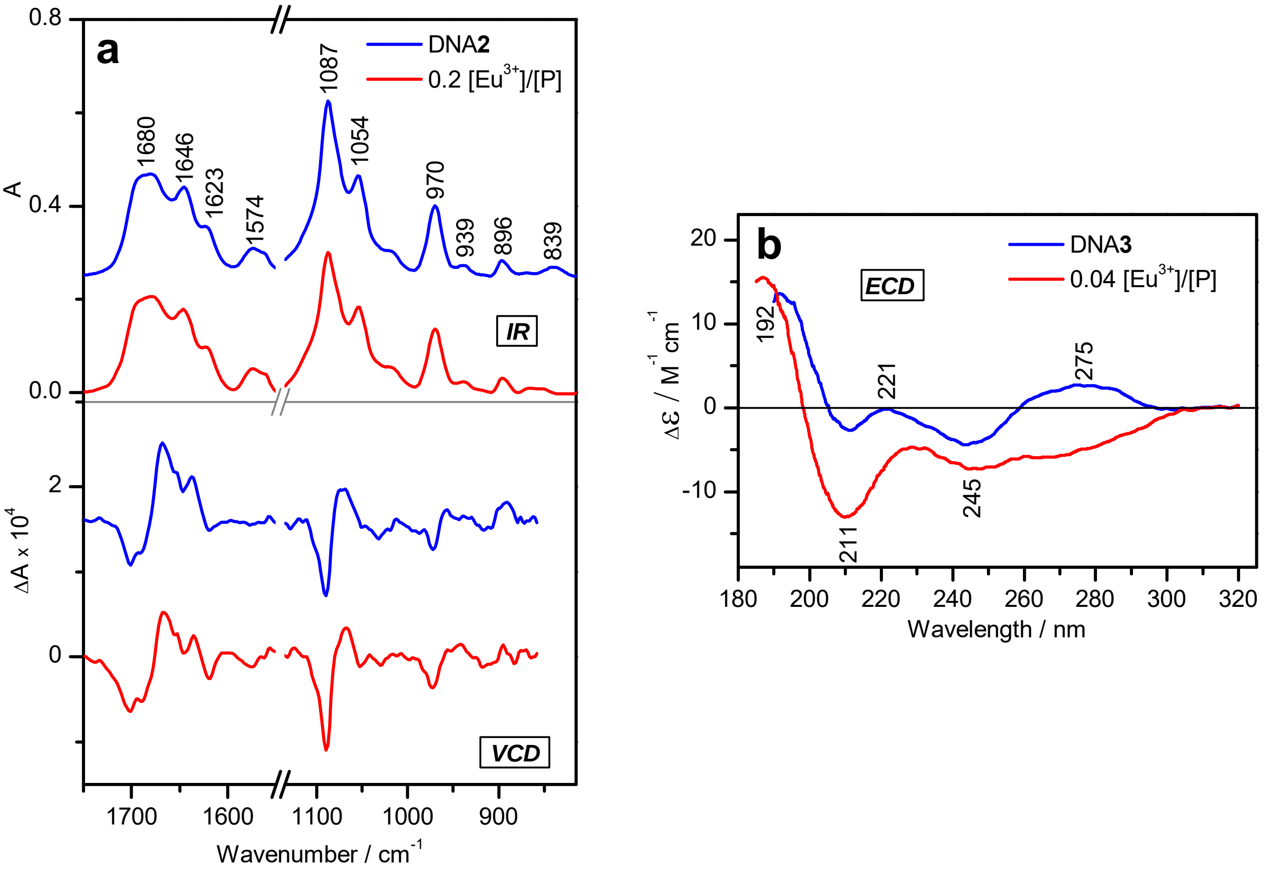

Figure S4. (a) IR (top) and VCD (bottom) spectra of blank DNA**2** (90 mM (P)) and in the presence of 18 mM of $EuCl_3$ (0.2 $[Eu^{3+}]$/[P]). The IR and VCD spectra are vertically shifted for clarity. **(b)** ECD spectra of blank DNA**3** (10 mM (P)) and in the presence of 0.4 mM of $EuCl_3$ (0.04 $[Eu^{3+}]$/[P]).

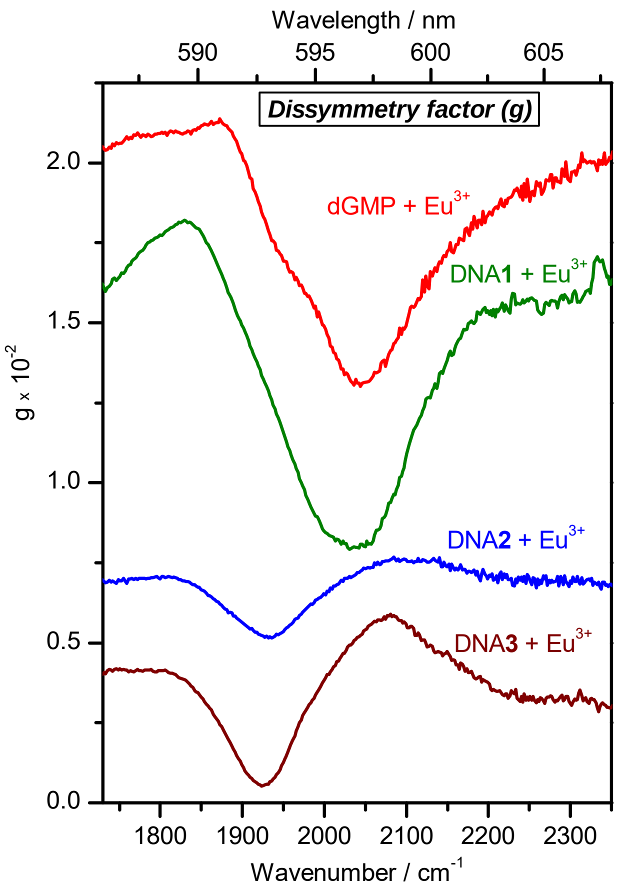

Figure S5. Dissymmetry factor (g) of dGMP and DNA complexes with Eu^{3+}. Dissymmetry factor was determined as $g = 2\times(I_L - I_R)/(I_L + I_R)$, where I_L and I_R represent the corresponding intensities of left- and right-circularly polarized emission. The spectra are vertically shifted for clarity.

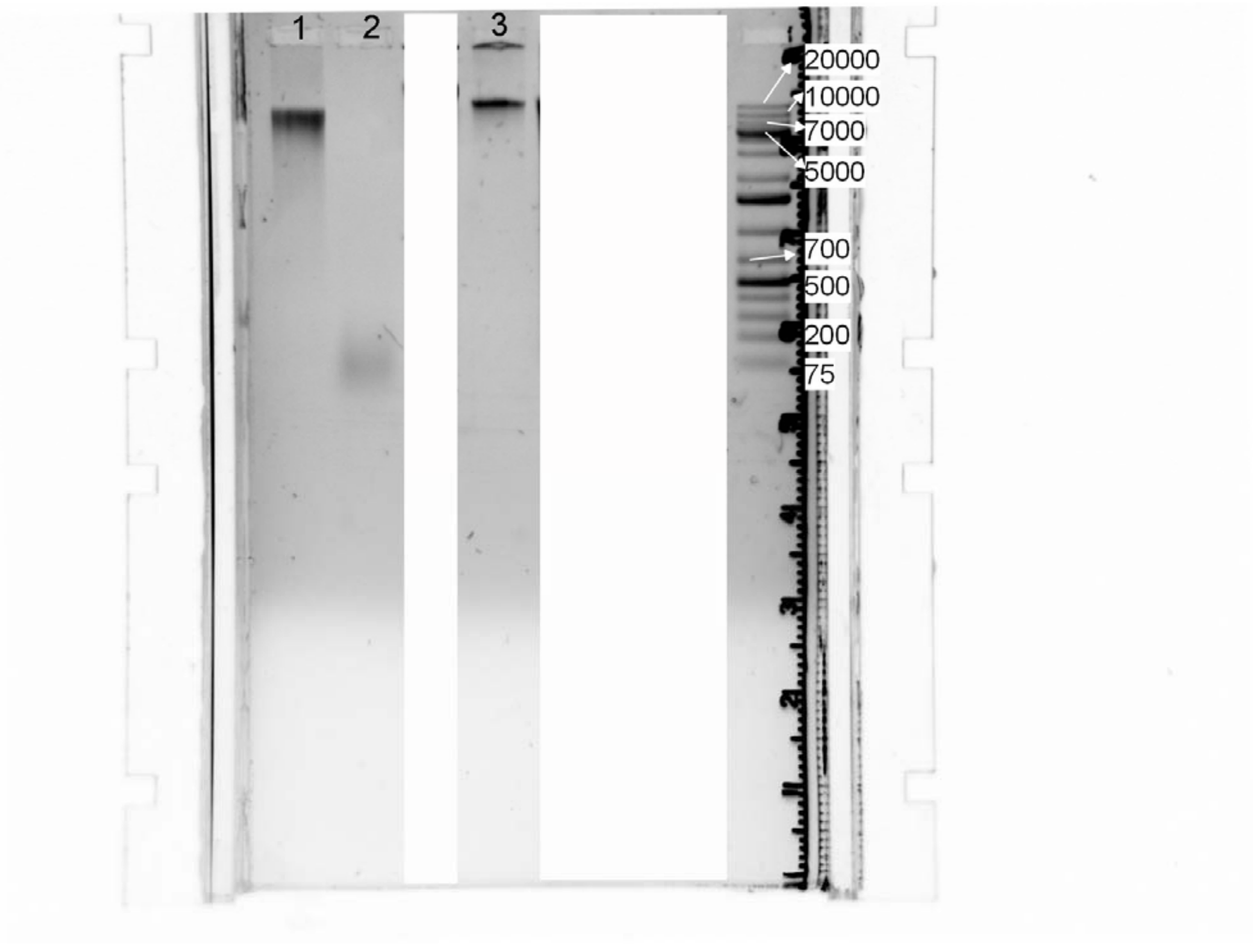

Figure S6. Gel-electrophoresis of DNA**1** (lane 2), DNA**2** (lane 1), and DNA**3** (lane 3). The molecular size marker is in the rightmost lane.

Table S1. Luminescence bands observed in MROA/MCPL spectra of $EuCl_3$ aqueous solution

Raman shift / cm^{-1}	Luminescence band / nm	Transition
1940	593	
1903	592	$^5D_0 \rightarrow {}^7F_1$
1848	590	
1845	590	
1759	587	
1726	586	$^5D_0 \rightarrow {}^7F_1$
1693	585	$^5D_1 \rightarrow {}^7F_3$
1670	584	
1658	583	
1521	579	$^5D_0 \rightarrow {}^7F_0$
894	558	
851	557	
790	555	
771	555	$^5D_1 \rightarrow {}^7F_2$
713	553	
708	553	
